# Inflammatory markers in intrahepatic cholangiocarcinoma: Effects of advanced liver disease

**DOI:** 10.1002/cam4.2373

**Published:** 2019-08-20

**Authors:** Cortlandt M. Sellers, Johannes Uhlig, Johannes M. Ludwig, Stacey M. Stein, Hyun S. Kim

**Affiliations:** ^1^ Section of Interventional Radiology, Department of Radiology and Biomedical Imaging Yale School of Medicine New Haven Connecticut; ^2^ Department for Diagnostic and Interventional Radiology University Medical Center Goettingen Goettingen Germany; ^3^ Department of Diagnostic and Interventional Radiology and Neuroradiology University Hospital Essen, University of Duisburg‐Essen Essen Germany; ^4^ Section of Medical Oncology, Department of Internal Medicine Yale School of Medicine New Haven Connecticut; ^5^ Yale Cancer Center, Yale School of Medicine New Haven Connecticut

**Keywords:** bile ducts, cholangiocarcinoma, intrahepatic, liver cirrhosis, lymphocytes, neutrophils, platelets

## Abstract

**Background:**

To investigate the neutrophil‐to‐lymphocyte ratio (NLR), platelet‐to‐lymphocyte ratio (PLR), and systemic immune‐inflammation index (SII) as prognostic biomarkers in intrahepatic cholangiocarcinoma (ICC) with a focus on viral hepatitis and liver status.

**Methods:**

In this retrospective cohort study, patients from the institutional cancer registry with ICC from 2005 to 2016 were stratified by treatment group. Baseline inflammatory markers were dichotomized at the median. Overall survival (OS) was assessed via Kaplan‐Meier curves and Cox proportional hazard models. Multiple patient, liver, and tumor factors were included in the multivariable analysis (MVA).

**Results:**

About 131 patients (median age 65 years, 52% male, 76% Caucasian) had a median OS of 13.0 months. Resection/interventional oncology with/without systemic therapy had improved survival vs systemic therapy alone in Child‐Pugh A patients (*P* < 0.01). In Child‐Pugh B/C patients, this survival difference became nonsignificant (*P* = 0.22). Increased NLR and SII were associated with decreased survival (*P* < 0.01), while dichotomized PLR was not (*P* = 0.3). On MVA, increased NLR remained an independent prognostic factor (HR 1.6, *P* < 0.05). In Child‐Pugh class A (n = 94), low‐NLR had higher OS vs high‐NLR (25.4 vs 12.2 months, *P* < 0.01). In Child‐Pugh class B/C (n = 28), NLR did not have a significant effect on median OS (low‐ vs high‐NLR: 6.7 vs 2.9 months, *P* = 0.2). Child‐Pugh class acted as an effect modifier on MVA for NLR (*P* = 0.0124).

**Conclusions:**

The NLR has a stronger impact as a prognostic marker in ICC over the PLR and SII. This survival effect is decreased in advanced liver disease.

## Graphical abstract

The neutrophil‐to‐lymphocyte ratio (NLR) has the strongest impact as a prognostic marker in intrahepatic cholangiocarcinoma (ICC) versus the platelet‐to‐lymphocyte ratio and systemic immune‐inflammation index, and increased NLR is associated with decreased survival in ICC. The NLR has decreased prognostic utility in patients with advanced liver disease.

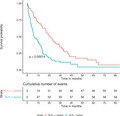

## 1 INTRODUCTION

Intrahepatic cholangiocarcinoma (ICC) is the second most common form of primary liver cancer,^1^ comprising 5%‐10% of cases,^2^ with an annual age‐standardized incidence rate in western countries of <1.5 cases per 100 000 persons.^3^ Major risk factors for ICC include primary sclerosing cholangitis, intrahepatic lithiasis, biliary tree anomalies, liver fluke infection, chronic liver disease, and cirrhosis.^4^ More recently, it has been confirmed that exposure to viral hepatitis,^5^ nonalcoholic fatty liver disease, and diabetes^6^ may also increase the risk of ICC development.

ICC carries a dismal prognosis, with 3‐ and 5‐year survival rates of 30% and 18%.^7^ Surgical resection is recommended whenever possible^8^; however, as few as 15% of patients may present with inoperable disease.^9^ For those who are not surgical candidates, systemic chemotherapy is the standard of treatment.^8^ Recently, interventional oncology (IO) therapies have been used in ICC for palliation^10^, ^11^ and as an adjuvant therapy.^12^, ^13^ Additional work has examined the feasibility of IO therapies in unresectable disease.^14^, ^15^, ^16^

Chronic inflammation and the immune response are integral to the development of cancers, including ICC.^17^, ^18^ The risk factors associated with ICC create a neoplasia‐prone environment through first liver injury and then inflammation, leading to the generation of free radicals and the stimulation of cytokines, chemokines, and other growth factors.^17^ Activation of cellular proliferation and increased cellular DNA synthesis may accelerate the rate of cellular mutations and advancement along the cancer pathway.^19^

The neutrophil‐to‐lymphocyte ratio (NLR) has been studied as a biomarker in multiple solid tumors, such as hepatocellular carcinoma, renal, breast, and metastatic melanoma.^20^, ^21^, ^22^, ^23^ Several researchers have also investigated the NLR in ICC^24^, ^25^, ^26^, ^27^; however, little work has been done on the relative importance of the NLR, the platelet‐to‐lymphocyte ratio (PLR), and the systemic immune‐inflammation index (SII). Furthermore, this study seeks to examine the interplay between inflammatory markers and underlying liver disease in ICC, with the hypothesis that chronic inflammation may affect the prognostic value of these biomarkers.

## 2 METHODS

### 2.1 Study population

The protocols and methods were conducted in compliance with the Health Insurance Portability and Accountability Act and were approved by the institutional review board. Patients diagnosed with ICC from 2005 to 2016 were retrospectively identified from the institutional cancer registry of a single urban academic center. Treatment allocation had been determined by a multi‐disciplinary tumor board. Exclusion criteria included individuals under the age of 18, incomplete treatment or survival data, and those with a histopathologic diagnosis of hepato‐cholangiocarcinoma.

### 2.2 Data acquisition

The variables reported by the cancer registry included age, gender, race/ethnicity, insurance status, marital status, American Joint Committee on Cancer (AJCC) staging, and basic treatment status. Furthermore, electronic medical record review was conducted to identify multiple other tumor and liver factors at the time of diagnosis along with the temporal sequence of treatments. Along with liver factors, baseline laboratory values were used to calculate Model for End‐Stage Liver Disease (MELD) and Child‐Pugh scores were missing from the medical record. The Charlson Comorbidity Index 28 (CCI) was calculated to quantify the burden of non‐ICC‐related disease. Viral hepatitis was defined via International Classification of Diseases 9th Edition codes as well as HBsAg and HCV Ab laboratory data and included patients at various stages of treatment, including the untreated.

Treatment allocation was first stratified into systemic therapy (chemotherapy and/or radiation), IO (comprised of transarterial chemoembolization, radioembolization, and thermal ablation), resection, and supportive therapy (palliative care or no treatment). Patients who received IO or resection following or in conjunction with systemic therapy were classified as IO and resection, respectively. “Nonsurgical treatment” consisted of IO and systemic therapies. Patients who received treatment were further reclassified into three groups: those who received resection or IO first with/without subsequent systemic therapy (Group 1); patients who received systemic therapy followed by resection or IO (Group 2); and patients who received systemic therapy alone (Group 3). Overall survival (OS) was defined as time from diagnosis to time of death, regardless of etiology.

Baseline pretreatment absolute neutrophil, lymphocyte, and platelet numbers were determined from complete blood count with differential values drawn within 30 days prior to treatment or 30 days prior to diagnosis (for supportive treatment patients only) and were used to calculate the various inflammatory markers. In instances where the absolute neutrophil count (ANC) or absolute lymphocyte count (ALC) were not reported in the electronic medical record, neutrophils were calculated by multiplying the white blood cell count by the differential percentage of the neutrophils and similarly for the lymphocytes. The NLR ratio was defined as ANC/ALC. The PLR ratio was defined as platelet count/ALC, and the SII was defined as ANC*platelet count/ALC.

### 2.3 Statistical analysis

Continuous variables were compared using the Wilcoxon rank sum test and categorical variables using the chi‐squared test. Kaplan‐Meier methods and log‐rank tests were used to estimate OS. In instances where survival curves crossed, the Wilcoxon test was employed to account for early survival losses. Inflammatory markers were dichotomized at the median for visualization purposes but were log‐transformed and evaluated as linear factors for all additional survival analyses. Predictors of OS were identified using univariate and multivariable Cox proportional hazard models. Factors that were significant on univariate models were included in the multivariable analysis (MVA) to address confounding. Separate MVA was modeled including all inflammatory markers (not reported). Among the biomarkers, only NLR retained statistical significance and was therefore considered the most relevant variable for further MVA modeling in order to avoid overfitting. A multiplicative interaction term between Child‐Pugh class and log‐transformed NLR (Child_Pugh*log_NLR) was included in multivariable Cox models to assess for heterogenous effects of NLR according to Child‐Pugh class. Patients with missing variables were conservatively excluded from analyses to account for bias.

All *P*‐values reported are two‐sided, with an alpha level of 0.05 considered statistically significant. Statistical analyses were completed using JMP Pro v.13.0.0 (SAS Institute Inc, Cary, NC), and GraphPad Prism v.7.0a for Mac (GraphPad Software, La Jolla, CA). Additional figure preparation was completed using R v.3.4.3 (R Core Development Team, Vienna, Austria).

## 3 RESULTS

### 3.1 Demographics

One hundred and thirty‐one patients (median age 65 years, 51.9% male, 75.6% Caucasian) met the inclusion criteria, with median follow‐up time of 12.0 months (IQR 4.5‐25.2 months) and 109 deaths during the study period (See Figure S1 for study design). Seventy‐seven percent of patients (n = 101) had confirmed histopathologic evidence of ICC. The majority of these biopsies were poorly or moderately‐to‐poorly differentiated (n = 58, 57.4%). Median CCI was 7.0 (IQR 5.0‐9.0), and median MELD was 8.0 (IQR 6.0‐11.0). 17.6% of patients (n = 23) had viral hepatitis, and 26% (n = 34) had cirrhosis. Seventeen patients had hepatitis C (74%), five had hepatitis B (22%), and one had coinfection with hepatitis B and hepatitis C (4%). The annual number of new ICC cases increased over time, from four in 2005 to 18 in 2016. Twelve patients were presented with AJCC Stage I disease (9.2%); 27 were Stage II (20.6%); 11 were Stage III (8.4%); and 56 patients were Stage IV (42.7%). At diagnosis, 72 patients (55.0%) had multifocal ICC, 64 patients (48.9%) had bilobar cholangiocarcinoma, and 60 patients (45.8%) had metastatic disease. Median tumor size was 6.5 cm (IQR 4.1‐8.8). Refer to Table 1 for additional baseline data.

**Table 1:** Patient, liver, and tumor factors by neutrophil‐to‐lymphocyte group

Factor		Entire cohort (n = 131)	NLR ≤ 3.95 (n = 66)	NLR > 3.95 (n = 65)	*P*‐value
Age		65.0 (57.0‐71.0)	62.0 (56.0‐70.3)	66.0 (59.0‐72.0)	0.0670
Male Gender		68 (51.9%)	37 (56.1%)	31 (47.7%)	0.3376
Race/Ethnicity					0.2509
Caucasian, non‐Hispanic		99 (75.6%)	47 (71.2%)	52 (80.0%)	
Black, Non‐Hispanic		16 (12.2%)	8 (12.1%)	8 (12.3%)	
Hispanic		10 (7.6%)	8 (12.1%)	2 (3.1%)	
Other/Unknown		6 (4.6%)	3 (4.6%)	3 (4.6%)	
Primary Insurance					0.2290
Private		75 (60.0%)	40 (66.7%)	35 (53.9%)	
Medicare		36 (28.8%)	13 (21.7%)	23 (35.4%)	
Medicaid		14 (11.2%)	7 (11.7%)	7 (10.8%)	
Charlson Comorbidity Index		7.0 (5.0‐9.0)	7.0 (5.0‐8.3)	8.0 (5.5‐10.0)	**0.0250**
Viral Hepatitis		23 (17.6%)	17 (25.8%)	6 (9.2%)	**0.0126**
Cirrhosis		34 (26.0%)	19 (28.8%)	15 (23.1%)	0.0838
Child‐Pugh Class					0.1043
A		94 (71.8%)	50 (75.8%)	44 (67.7%)	
B		26 (19.8%)	10 (15.2%)	16 (24.6%)	
C		2 (1.5%)	0 (0.0%)	2 (3.1%)	
Missing/Unknown		9 (6.9%)	6 (9.0%)	3 (4.6%)	
MELD Score		8.0 (6.0‐11.0)	7.0 (6.0‐9.0)	8.0 (6.0‐13.0)	0.0643
AJCC Stage					0.3482
I		12 (15.3%)	9 (13.4%)	3 (4.6%)	
II		27 (20.6%)	15 (22.7%)	12 (18.5%)	
III		11 (8.4%)	5 (7.6%)	6 (9.2%)	
IV		56 (42.7%)	27 (40.9%)	29 (44.6%)	
Missing/Unknown		25 (19.1%)	10 (15.2%)	15 (23.1%)	
Tumor Size (cm)		6.5 (4.1‐8.8)	5.8 (4.0‐7.2)	7.5 (5.4‐12.0)	**0.0007**
Tumor Location					**0.0104**
Unilobar		65 (50.4%)	40 (60.6%)	25 (38.5%)	
Bilobar		64 (49.6%)	25 (37.9%)	39 (60.0%)	
Multifocal Disease		72 (55.0%)	36 (54.6%)	36 (55.4%)	0.8173
Vascular Invasion		33 (25.2%)			
Metastatic Disease		60 (45.8%)	37 (40.9%)	33 (50.8%)	0.3913
ANC (*10^3^/µL)		5.3 (3.5‐7.7)	4.0 (3.0‐5.3)	7.1 (5.3‐10.2)	**<0.0001**
Patient characteristics by NLR					
ALC (*10^3^/µL)		1.4 (1.0‐1.8)	1.7 (1.3‐2.2)	1.2 (0.8‐1.4)	**<0.0001**
Platelets (*10^3^/µL)		229 (161‐298)	210.5 (149.8‐280.5)	253.0 (162.0‐324.0)	0.0885
NLR		3.95 (2.36‐6.19)	2.37 (1.81‐3.09)	6.19 (4.77‐12.68)	**<0.0001**
PLR		156 (111‐226)	118 (86‐152)	214 (162‐290)	**<0.0001**
SII		867 (455‐1422)	462 (326‐746)	1406 (970‐2243)	**<0.0001**
Treatment					**0.0381**
Resection		28 (21.4%)	20 (30.3%)	8 (12.3%)	
IO		16 (12.2%)	8 (12.1%)	8 (12.3%)	
Systemic Therapy		65 (49.6%)	31 (47.0%)	34 (52.3%)	
Supportive Therapy		22 (16.8%)	7 (10.6%)	15 (23.1%)	

Data presented as median (IQR) or N (%).

Abbreviations: AJCC, American Joint Committee on Cancer; ALC, absolute lymphocyte count; ANC, absolute neutrophil count; IO, interventional oncology; MELD, Model for End‐stage Liver Disease; NLR, neutrophil‐to‐lymphocyte ratio; PLR, platelet‐to‐lymphocyte ratio; SII, systemic immune‐inflammation index.

Bold indicates *P*‐values falling below the level of significance (*P* < 0.05).

### 3.2 Treatment allocation

Sixteen patients received IO (12.2%), 65 patients received systemic therapy (49.6%), 28 patients underwent resection (21.4%), and 22 patients received supportive therapy (16.8%). Thirty‐five patients received resection or IO with/without subsequent systemic therapy (Group 1, 26.7%); nine patients received systemic therapy followed by IO or resection (Group 2, 6.9%); and 70 patients received systemic therapy alone (Group 3, 49.6%).

Patients who received surgical treatment were more likely to be younger, Child‐Pugh class A, AJCC Stage I, have solitary tumors, unilobar disease, and to not have metastases (*P* < 0.01). Supportive therapy patients had higher MELD and CCI scores than nonsurgical or surgical treatment (*P* < 0.01). Gender, race, insurance, viral hepatitis status, and the presence or absence of cirrhosis did not affect the likelihood of receiving treatment (*P* > 0.05). Surgical treatment was associated with lower median tumor size (4.2 cm) vs nonsurgical treatment (7.0 cm) or supportive care (7.5 cm, *P* = 0.0006).

### 3.3 Overall survival and recurrence

Median OS for the entire cohort was 13.0 months (95% CI: 9.2‐16.8 months) and was highest in resection (median OS 43.8 months), followed by IO (33.1 months), systemic therapy (11.0 months), and supportive therapy (1.3 months, overall *P* < 0.0001). Improved survival was also seen on MVA for IO as compared to systemic therapy (HR 8.32; 95% CI: 2.70‐29.81, *P* = 0.0001).

**Figure 1:**
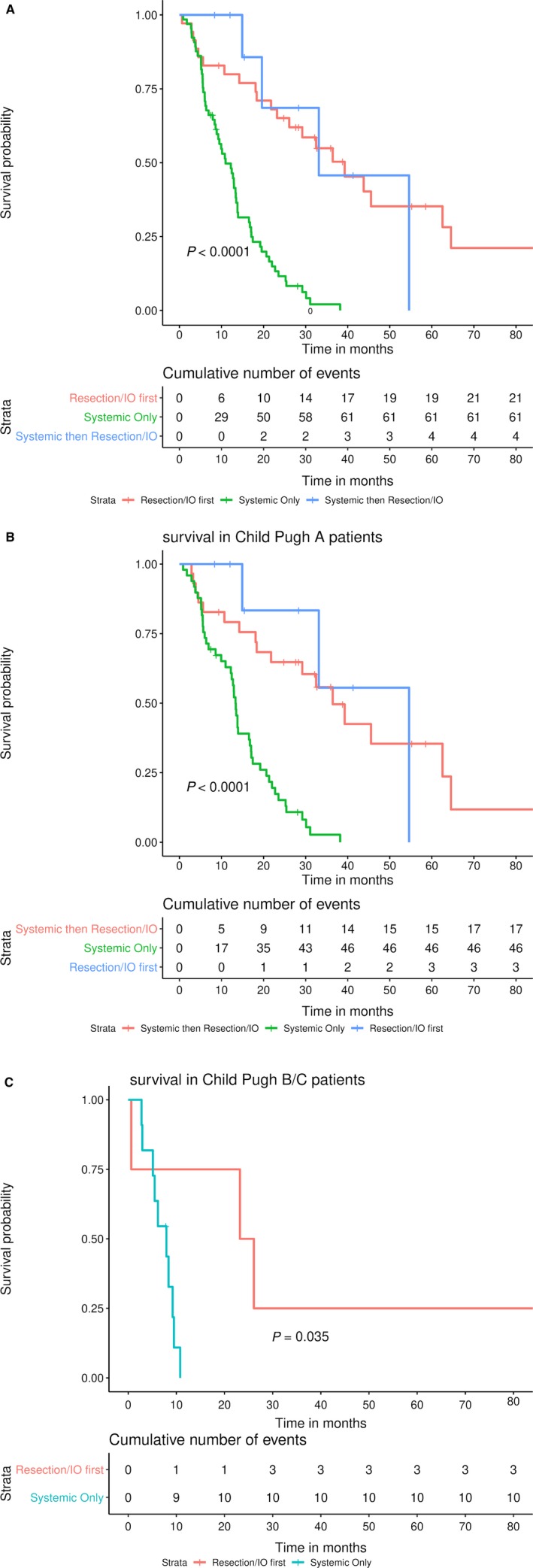
Overall survival by treatment group. A, Entire cohort; B, Child‐Pugh A patients; C, Child‐Pugh B patients

OS was highest in Group 1 (39.3 months), followed by Group 2 (33.1 months) and Group 3 (11.0 months, *P* < 0.01) (Figure 1A). In patients with Child‐Pugh A disease, Group 2 demonstrated the highest survival (Group 1 vs 2 vs 3: median OS 36.4 vs 54.6 vs 13.3 months, *P* < 0.0001) (Figure 1B). In patients with Child‐Pugh class B disease, there was a nonsignificant trend toward improved survival for patients in Group 1 vs Group 3 (*P* = 0.22) (Figure 1C).

### 3.4 Prognostic factors

Factors significant on univariate analysis (*P* < 0.05) included age, insurance, CCI, Child‐Pugh stage, MELD score, tumor size, tumor location, multifocal disease, extrahepatic metastases, AJCC staging, treatment allocation, ANC, ALC, and log‐transformed NLR, SII, and PLR (Table 2). Nonsignificant factors included gender, race, viral hepatitis status, vascular invasion, the presence or absence of cirrhosis, and platelet counts. Significant factors (*P* < 0.05) on MVA included treatment allocation, insurance type, and log‐transformed NLR (Table 3).

**Table 2:** Significant and nonsignificant prognostic markers on univariate analysis

Factor	HR	Lower 95%	Upper 95%	*P*‐value
Age	1.02	1.001	1.038	0.0391
Male gender	1.20	0.820	1.764	0.3498
Race				
Caucasian (reference)				
African American	0.88	0.441	1.600	0.6972
Hispanic	1.08	0.525	1.991	0.8166
Other/Unknown	2.19	0.845	4.646	0.0995
Insurance				
Private (reference)				
Medicare	1.90	1.207	2.930	0.0062
Medicaid	1.54	0.809	2.726	0.1773
Charlson Comorbidity Index	1.26	1.172	1.355	<0.0001
Cirrhosis	1.54	0.978	2.361	0.0618
Viral hepatitis	0.93	0.517	1.601	0.7935
Child Pugh class				
A (reference)				
B/C	2.84	1.758	4.437	<0.0001
MELD	1.08	1.046	1.102	<0.0001
AJCC stage				
I (reference)				
II	2.14	0.934	5.543	0.0727
III	2.58	0.900	7.598	0.0770
IV	6.39	2.88	16.49	<0.0001
Tumor size	1.11	1.045	1.177	0.0005
Tumor location				
Unilobar (reference)				
Bilobar	2.30	1.542	3.437	<0.0001
Multifocal Tumor	2.31	1.547	3.486	<0.0001
Vascular invasion	1.74	0.906	3.485	0.0968
Extrahepatic metastases	2.97	1.970	4.514	<0.0001
Treatment				
Resection (reference)				
IO	1.76	0.703	4.113	0.2156
Systemic	5.62	3.070	11.063	<0.0001
Supportive	21.71	10.674	45.836	<0.0001
ANC (continuous)	1.14	1.067	1.2016	0.0001
ALC (continuous)	0.58	0.403	0.811	0.0013
Platelets (continuous)	1.00	0.998	1.002	0.8154
log(NLR)	2.11	1.555	2.847	<0.0001
log(PLR)	1.43	1.007	2.036	0.0454
log(SII)	1.38	1.106	1.732	0.0043

Abbreviations: AJCC, American Joint Committee on Cancer; ALC, absolute lymphocyte count; ANC, absolute neutrophil count; IO, interventional oncology; MELD, Model for End‐stage Liver Disease; NLR, neutrophil‐to‐lymphocyte ratio; PLR, platelet‐to‐lymphocyte ratio; SII, systemic immune‐inflammation index.

**Table 3:** Significant and nonsignificant prognostic markers on multivariate analysis

Factor	HR	Lower 95%	Upper 95%	*P*‐value
Age	0.98	0.942	1.026	0.4281
Insurance				
Private (reference)				
Medicare	2.46	1.251	4.851	**0.0093**
Medicaid	1.31	0.535	3.001	0.5366
Charlson Comorbidity Index	1.13	0.855	1.466	0.3694
Child‐Pugh class				
A (reference)				
B/C	0.62	0.190	2.031	0.4246
MELD	1.03	0.971	1.092	0.2851
AJCC stage				
I (reference)				
II	1.40	0.442	5.102	0.5749
III	1.46	0.322	6.786	0.6234
IV	2.16	0.535	9.457	0.2819
Tumor size	1.06	0.956	1.170	0.2562
Tumor location				
Unilobar (reference)				
Bilobar	1.54	0.776	3.036	0.2154
Multifocal Tumor	1.34	0.734	2.525	0.3430
Extrahepatic metastases	0.68	0.166	2.914	0.5946
Treatment Allocation				
Resection (reference)				
IO	0.95	0.263	3.273	0.9406
Systemic	4.40	1.550	13.360	**0.0051**
Supportive	54.5	10.952	286.738	**<0.0001**
log(NLR)	1.58	1.010	2.512	**0.0469**
Interaction term: Log(NLR)*Child‐Pugh score				**0.0124**

Abbreviations: AJCC, American Joint Committee on Cancer; HR, hazard ratio; IO, interventional oncology; MELD, Model for End‐stage Liver Disease; NLR, neutrophil‐to‐lymphocyte ratio; SII, systemic immune‐inflammatory index.

Bold indicates *P*‐values falling below the level of significance (*P* < 0.05).

### 3.5 Inflammatory marker selection

Median ANC for the cohort was 5.3 × 10^3^/L (IQR 3.5‐7.7), with median ALC of 1.4 × 10^3^/L (IQR 1.0‐1.8), and median platelets of 229 × 10^3^/L (IQR 161‐298). When dichotomized at the median, increased neutrophils were associated with poorer survival (median OS 9.2 months, 95% CI: 5.3‐13.4 months) vs decreased neutrophils (median OS 16.8 months, 95% CI: 12.4‐22.7 months, *P* = 0.0126). Conversely, patients with an ALC above the median had a higher median OS of 16.5 months (95% CI: 9.9‐23.5 months) vs patients with lymphocytes below or equal to the median (median OS 10.1 months, 95% CI: 5.2‐13.8 months, *P* = 0.0222). Increased vs decreased platelets did not appear to affect survival (median OS 14.2 months, 95% CI: 9.5‐19.5 months; vs 12.2 months, 95% CI: 6.4‐13.9 months, *P* = 0.5922). Similarly, on univariate proportional hazards analyses, increased neutrophils and decreased lymphocytes were associated with decreased survival (*P* < 0.05), while there was no association between platelet count and survival (*P* > 0.05).

**Figure 2:**
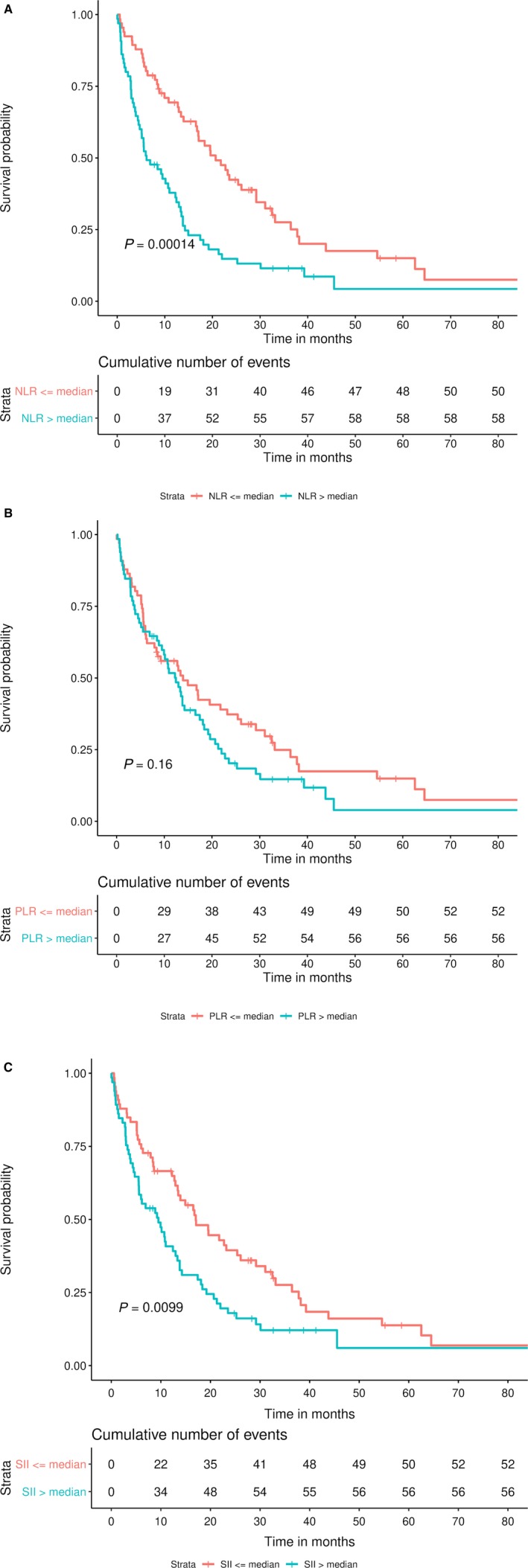
Overall survival by inflammatory marker groups. A, Neutrophil‐to‐lymphocyte ratio. B, Platelet‐to‐lymphocyte ratio. C, Systemic immune‐inflammation index

Median NLR for the cohort was 3.95 (IQR 2.36‐6.19), with median PLR of 156.43 (IQR 110.6‐225.6) and median SII of 867.4 (455.0‐1421.5). Correlation between inflammatory markers ranged from good (Spearman's *ρ* = 0.65) to very strong (ρ = 0.84). Decreased NLR and SII were associated with increased survival (*P* < 0.01) (Figure 2A,B), while dichotomized PLR was not (*P* = 0.3) (Figure 2C). ANC, ALC, log‐transformed PLR, and log‐transformed SII were not independent prognostic factors on MVA (*P* > 0.05). However, an increase in log‐transformed NLR continued to be associated with decreased survival (HR 1.96, *P* = 0.0001) on MVA. Given that the NLR was the only inflammatory marker to remain significant on MVA, the NLR was used for further subgroup analyses.

### 3.6 Neutrophil‐to‐lymphocyte ratio

Patients with NLR greater than the median had larger median tumor size (7.5 vs 5.8 cm, *P* = 0.0007) and were more likely to have viral hepatitis, bilobar disease, or to receive systemic or supportive therapy (*P* < 0.05). Patients across NLR groups were similar in terms of age, gender, race/ethnicity, insurance status, cirrhosis status, Child‐Pugh score, MELD score, and AJCC stage. The low‐NLR group had improved median OS (NLR ≤ 3.96, 20.7 months) vs the high‐NLR group (NLR > 3.96, OS 6.2 months). When stratified by treatment allocation, the NLR remained a significant factor in patients treated with systemic therapy (NLR ≤ 3.96 vs NLR > 3.96; median OS 13.4 vs 9.2 months; log‐rank test *P* = 0.0130). Higher survival was also seen in resection (n = 28; low‐ vs high‐NLR; median OS 36.4 vs 42.4 months; *P* = 0.83) and IO (n = 16; low‐ vs high‐NLR; median OS 33.1 vs 10.7 months; *P* = 0.0674), although this did not reach statistical significance.

### 3.7 Liver status and NLR

**Figure 3:**
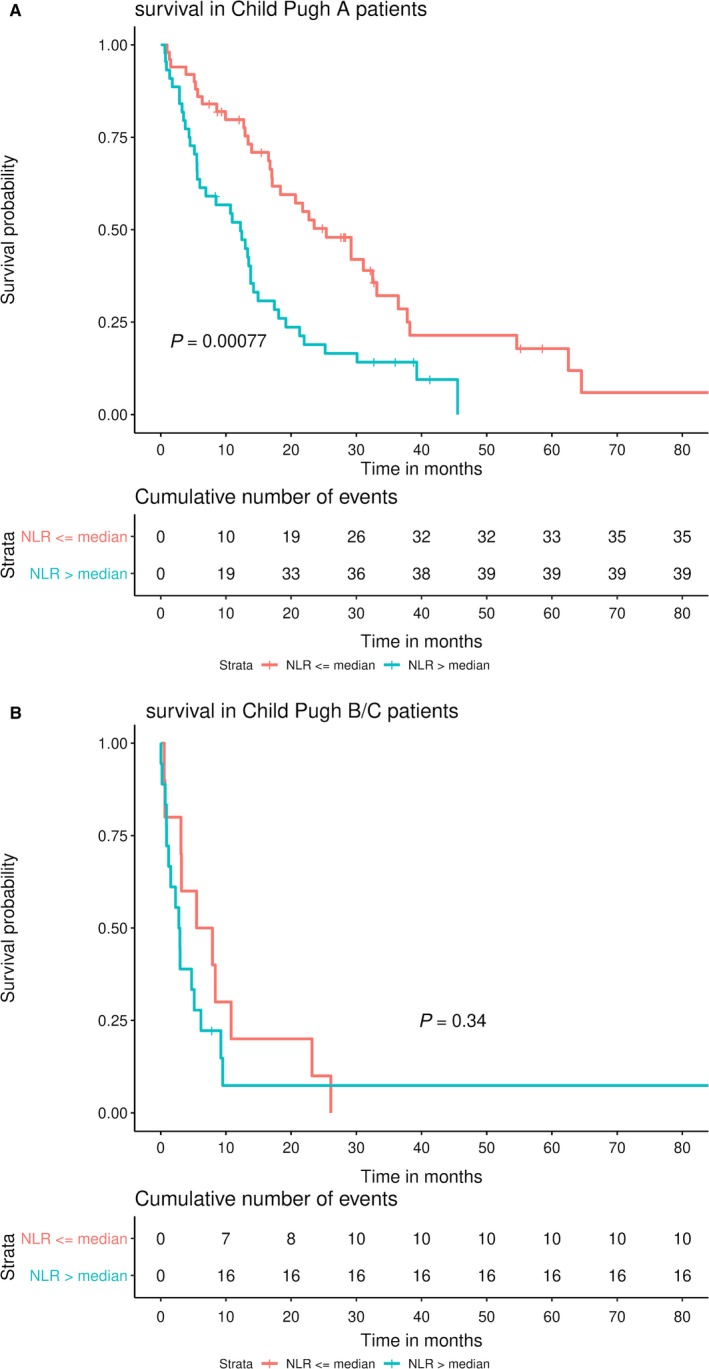
Overall survival by neutrophil‐to‐lymphocyte ratio and Child‐Pugh status. A, Child‐Pugh A patients. B, Child‐Pugh B patients

Patients with Child‐Pugh A disease had higher median ALC (*P* < 0.05), whereas patients with Child‐Pugh B/C disease had higher comorbidity indices (median 10.0 vs 6.5, *P* < 0.0001), MELD scores (13.0 vs 7.0, *P* < 0.0001), and higher rates of cirrhosis (*P* < 0.05). Patients with Child‐Pugh A disease also had higher rates of resection (22.4% vs 10.7%), IO (16.0% vs 3.6%), and systemic therapy (52.1% vs 39.3%) and lower rates of palliative therapy (8.5% vs 46.4%, overall *P* = 0.0001) as compared to Child‐Pugh B/C patients. Across both Child‐Pugh groupings, patients had similar distribution of age, gender, race/ethnicity, insurance, viral hepatitis, AJCC stage, tumor size, bilobar tumor burden, vascular invasion, metastatic disease, ANC, platelets, NLR, PLR, and SII and (*P* > 0.05). In Child‐Pugh class A (n = 94), low‐NLR had higher OS vs high‐NLR (25.4 vs 12.2 months, *P* < 0.01) (Figure 3A). In Child‐Pugh class B/C (n = 28), NLR did not have a significant effect on survival (low‐ vs high‐NLR: 6.7 vs 2.9 months, *P* = 0.2) (Figure 3B).The interaction term Child‐Pugh class*log(NLR) was also significant on MVA (*P* = 0.0124), demonstrating Child‐Pugh class to act as an effect modifier for NLR.

## 4 DISCUSSION

Intrahepatic cholangiocarcinoma is the second most common form of primary liver cancer^1^ and carries a very poor prognosis. ICC risk factors, including primary sclerosing cholangitis and intrahepatic lithiasis, predispose toward cancer development through injury, immune system activation, and cellular proliferation. This study sought to clarify the significance of three circulatory inflammatory markers and to examine the effect of chronic liver disease on their prognostic value.

During the study period, annual cases of newly diagnosed ICC increased from four in 2005 to 18 in 2016, a trend which is reflected on both a national^29^, ^30^ and a global scale.^4^ It is unclear if this is due to a true increase in incidence or to a possible change in referral patterns or increased rates of early detection. MVA demonstrated that patients treated with IO had improved survival vs those treated with systemic therapy alone. Furthermore, patients treated with resection or IO therapies with and without systemic therapy had greater survival than patients treated with systemic therapy alone. While this survival difference was also seen on subgroup analyses in patients with Child‐Pugh A disease, it became a nonsignificant trend among patients with Child‐Pugh B disease. This may in part be due to the smaller numbers of Child‐Pugh B patients included in this study (n = 26).

We observed strong correlation among the NLR, PLR, and SII; however, only the NLR remained an independent predictor of survival on MVA, where increased NLR was associated with decreased survival. This survival difference was also seen on subgroup analyses in patients who received systemic therapy, and there was a trend toward improved survival with decreased NLR in the surgery and IO treatment groups. Although this trend was nonsignificant, this may have been due to the smaller numbers in the surgery and IO subgroups.

Child‐Pugh status was demonstrated to be an effect modifier of the NLR. For patients with none‐to‐mild liver disease (Child‐Pugh A), the association between increased NLR and poorer survival remained significant. For those with moderate liver disease (Child‐Pugh B), however, there was no significant survival difference across NLR groups. It should be noted that the Child‐Pugh A and Child‐Pugh B groups in our cohort had similar distribution of inflammatory markers. Perhaps, in cases of advanced chronic inflammation, the immune system becomes fatigued, and the rates of increased neutrophils may be fewer. Furthermore, in light of the low numbers of Child‐Pugh class B patients present in this cohort, there may be smaller effects and nuances that are being missed due to low power.

### 4.1 Interventional oncology

It is thought that both transarterial chemoembolization (TACE)^10^ and Y‐90^11^ are safe and effective as palliative therapies for unresectable ICC. TACE has been used successfully as adjuvant therapy alongside chemotherapy^12^ and surgical resection.^13^ In a study comparing resection with TACE, Scheuermann et al^31^ found that there was no survival benefit for resection vs TACE in patients with positive lymph nodes or positive resection margins after surgery. Radiofrequency ablation has also been shown to prolong survival in inoperable ICC, particularly in tumors <5 cm.^15^, ^32^ In our study, we found that IO was associated with a survival benefit compared to systemic therapy. Collectively, this evidence suggests that IO therapies may be an area of future study for treatment of ICC, particularly in patients with comorbidities that prevent them from being surgical candidates or who are unable to tolerate the side effects of systemic chemotherapy.

### 4.2 Inflammation and ICC

Intrahepatic cholangiocarcinoma is a very heterogenous tumor, with multiple morphologies on gross,^33^ cellular,^34^ and molecular^35^ levels. Risk factors for ICC activate the inflammatory response, leading to increased cholangiocyte proliferation and the production of free radicals and multiple other cytokines and chemicals which continue the cycle of injury and growth,^17^, ^19^ creating an ideal environment for carcinogenesis.^18^, ^36^

As available knowledge about circulating inflammatory biomarkers grows, it is important to determine which markers carry the most prognostic value. Of the markers studied here, the NLR was most useful. Similarly, Ha and colleagues^37^ studied the NLR, PLR, and SII as well as soluble programmed cell death ligand‐1 in 158 patients with advanced biliary tract cancers, and concluded that only NLR and soluble PD‐L1 were independent prognostic factors.

Increased NLR ratios have been associated with poor prognosis following surgery^24^, ^27^, ^38^ and chemotherapy.^25^, ^27^ The NLR has been a component of potential prognostic systems^39^ and nomograms for the prediction of resection futility.^40^ A recent meta‐analysis comprising 26 studies and 4461 patients concluded that while increased NLR indicated a poor prognosis in primary liver cancers, subgroup analyses suggested that the predictive role of NLR in cholangiocarcinomas might be limited.^41^ Of note, only 29 of these 4461 patients had confirmed ICC.

The exact mechanism of how the NLR ratio affects survival is unclear. Lin et al^26^ examined 102 patients with ICC and saw that higher PD‐1+CD4+ and PD‐1+CD8+ T cells were found in the high‐NLR group while higher amounts of IFN+CD4+ and IFN+CD8+ T cells were seen in the low‐NLR group. Furthermore, the high‐NLR group experienced an increased density of tumor‐infiltrating CD3+T cells. However, in spite of these findings, the strengths of the NLR over PLR and SII^37^ suggest that it may be the neutrophil component and not the lymphocytic component which drives the effect of the NLR.

Given the vast heterogeneity of ICCs, further work is required to better understand the differences between patients with an increased NLR and those with a lower NLR on a cellular and molecular level. The decreased effectiveness of the NLR in advanced liver disease seen in this cohort suggests that a state of chronic inflammation not directly subject to the cancer may also affect how the body responds to the tumor. Perhaps in cases of significant or advanced chronic inflammation, the immune system becomes fatigued, and the rates of increased neutrophils may be fewer.

### 4.3 Limitations

Although our sample size is a limiting factor in subgroup analyses, our cohort was diverse and well‐characterized among gender, race, and treatment modalities. We limited the NLR, PLR, and SII values to within 30 days prior to ICC treatment or diagnosis (for supportive therapy patients) in order to improve standardization, as our data relied on archived records. Furthermore, this study lacks information whether patients with cirrhosis and viral hepatitis also suffered from portal hypertension or hypersplenism. However, only platelet counts should be directly affected by these sequelae of liver disease. As this study has been conducted among a United States (Western) population, results may not be globally generalizable.

## 5 CONCLUSION

The NLR ratio has the strongest impact as a prognostic marker in ICC versus the PLR and SII, and increased NLR is associated with decreased survival in ICC. This increased survival is modulated by liver status, suggesting that the interplay between acute tumor‐associated inflammation and chronic liver parenchymal dysfunction may further affect survival.

## CONFLICT OF INTEREST

The authors have no conflicts of interest to report. The authors certify that they have no affiliations with or involvement in any organization or entity with any financial interest in the subject matter or materials discussed in this manuscript. Hyun S. Kim served on Advisory boards for Boston Scientific and SIRTex.

## AUTHOR CONTRIBUTIONS

Cortlandt M. Sellers: Conceptualization, data curation, formal analysis, writing—original draft; writing—reviewing and editing, final approval. Johannes Uhlig: Formal analysis and writing—reviewing and editing, final approval. Johannes M. Ludwig: Formal analysis and writing—reviewing and editing, final approval. Stacey M. Stein: Formal analysis and writing—reviewing and editing, final approval. Hyun S. Kim: Conceptualization, formal analysis, and writing—reviewing and editing, Resources, Funding, Supervision, final approval.
