# Supplementary figures and images for: Inflammatory markers in intrahepatic cholangiocarcinoma: Effects of advanced liver disease
Source: Cancer Med. 2019 Aug 20;8(13):5916–29. doi: 10.1002/cam4.2373 (PMC6792510; doi:10.1002/cam4.2373)

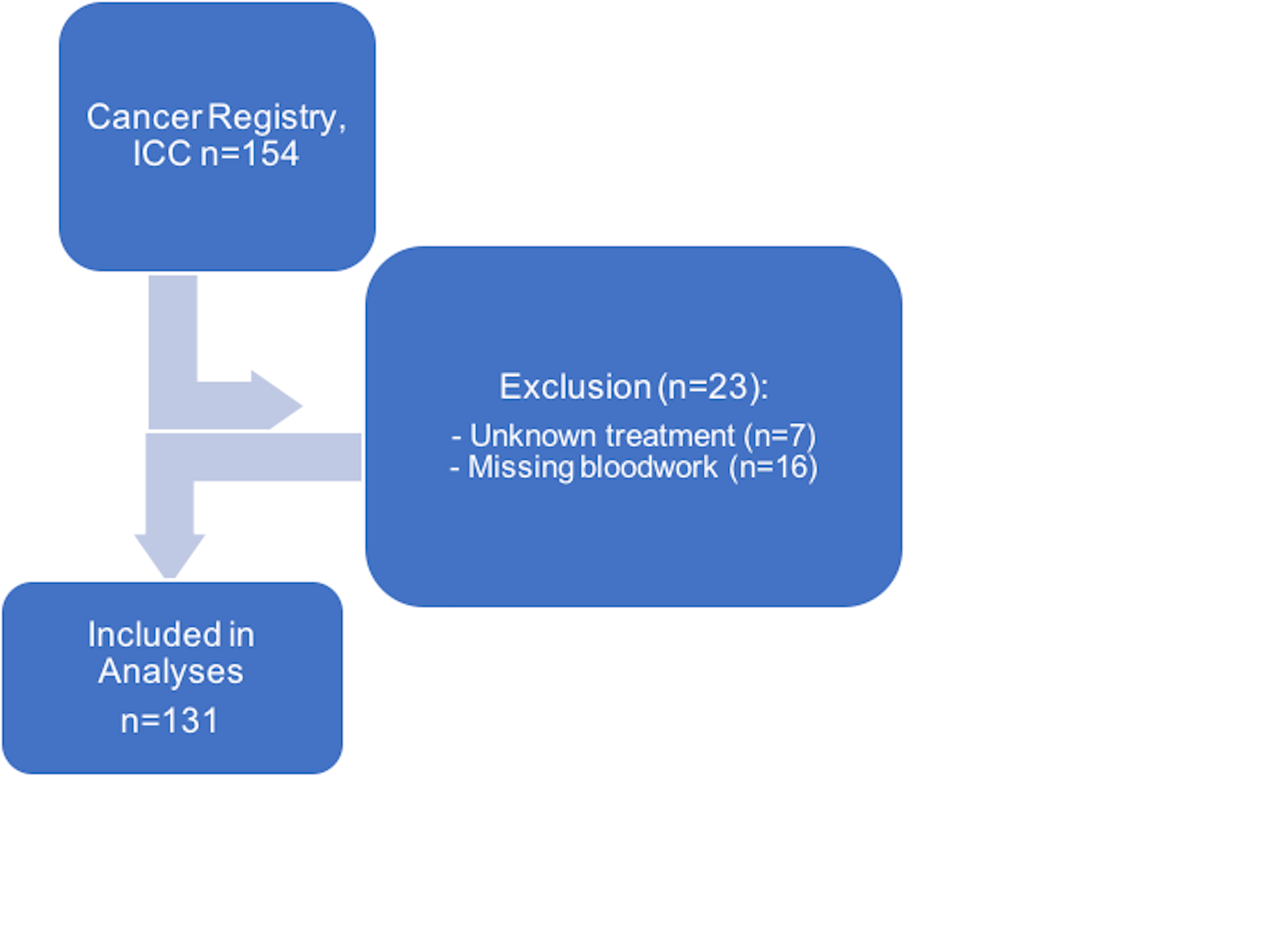

Supplement: Supplementary file 1 [file CAM4-8-5916-s001.tiff]
